# A modified standardized nine hole peg test for valid and reliable kinematic assessment of dexterity post-stroke

**DOI:** 10.1186/s12984-019-0479-y

**Published:** 2019-01-14

**Authors:** Gudrun M Johansson, Charlotte K Häger

**Affiliations:** Department of Community Medicine and Rehabilitation; Physiotherapy, Umeå University, Umeå, Building 15, Umeå University, SE-901 87 Umeå, Sweden

**Keywords:** Stroke, Upper extremity, Clinical laboratory techniques, Outcome assessment

## Abstract

**Background:**

Impairments in dexterity after stroke are commonly assessed by the Nine Hole Peg Test (NHPT), where the only outcome variable is the time taken to complete the test. We aimed to kinematically quantify and to compare the motor performance of the NHPT in persons post-stroke and controls (discriminant validity), to compare kinematics to clinical assessments of upper extremity function (convergent validity), and to establish the within-session reliability.

**Methods:**

The NHPT was modified and standardized (S-NHPT) by 1) replacing the original peg container with an additional identical nine hole pegboard, 2) adding a specific order of which peg to pick, and 3) specifying to insert the peg taken from the original pegboard into the corresponding hole of the target pegboard. Eight optical cameras registered upper body kinematics of 30 persons post-stroke and 41 controls during the S-NHPT. Four sequential phases of the task were identified and analyzed for kinematic group differences. Clinical assessments were performed.

**Results:**

The stroke group performed the S-NHPT slower (total movement time; mean diff 9.8 s, SE diff 1.4), less smoothly (number of movement units; mean diff 0.4, SE diff 0.1) and less efficiently (path ratio; mean diff 0.05, SE diff 0.02), and used increased scapular/trunk movements (acromion displacement; mean diff 15.7 mm, SE diff 3.5) than controls (*P* < 0.000, *r* ≥ 0.32), indicating discriminant validity. The stroke group also spent a significantly longer time grasping and releasing pegs relative to the transfer phases of the task compared to controls. Within the stroke group, kinematics correlated with time to complete the S-NHPT and the Fugl-Meyer Assessment (r_s_ 0.38–0.70), suggesting convergent validity. Within-session reliability for the S-NHPT was generally high to very high for both groups (ICCs 0.71–0.94).

**Conclusions:**

The S-NHPT shows adequate discriminant validity, convergent validity and within-session reliability. Standardization of the test facilitates kinematic analysis of movement performance, which in turn enables identification of differences in movement control between persons post-stroke and controls that may otherwise not be captured through the traditional time-based NHPT. Future research should ascertain further psychometric properties, e.g. sensitivity, of the S-NHPT.

## Background

Impaired upper limb dexterity is evident as in many as 45–70% of the stroke victims one year post-stroke [1, 2]. Such impairment is often evaluated in clinics by performance of the Nine Hole Peg Test (NHPT) [3], which is a frequently used dexterity task in many clinical populations [4–7]. The NHPT equipment consists of a container with nine small pegs and a target pegboard with nine holes. Performance of the NHPT requires the pegs to be picked up from the container one-by-one unimanually and transferred and inserted into the holes of the pegboard until it is filled, upon which the pegs are returned unimanually to the container. The test is performed as quickly as possible and the only outcome variable is the total time to complete the task. Consequently, motor performance is currently not analyzed during the NHPT despite potentially providing valuable information relating to upper limb dexterity, especially among persons with a neurological dysfunction.

Among persons with stroke, the NHPT is considered reliable [8], valid [7, 9, 10], and sensitive to change [7, 10, 11]. Nevertheless, and despite overall good test-retest reliability post-stroke, low test-retest reliability has been found in persons post-stroke who have spasticity in the affected hand [8]. Further, the measurement errors are large; the minimal detectable change of the NHPT is estimated to 33 s for an individual post-stroke, and even doubled in the presence of spasticity [8]. The measurement properties of computer-assisted assessments of NHPT in virtual environments have been investigated with promising results [12, 13]. However, high intra-subject variation indicates that haptic and virtual reality technologies are more demanding for a stroke population and for instance require more practice trials prior to the actual test than when performing a conventional NHPT.

Advantages of the NHPT include the simple, cheap and easily portable equipment as well as the test being easy to administer and time-efficient [7, 10]. There are, however, some drawbacks when testing persons post-stroke. First, the outcome score of the test is based solely on the time for task accomplishment [14]. Hence, a time reduction of the NHPT in rehabilitation of a person post-stroke may represent either a true motor recovery (i.e. performing movement patterns in a similar way as before the stroke) or compensation (performing different movement patterns than prior to the stroke) [15]. Compensatory strategies are common during upper limb tasks post-stroke, and thus plausible in a fine manipulative task like the NHPT. Secondly, the current NHPT test procedure may provide unreliable results for repeated measures or group comparisons as there is no standardized procedure with regard to the order in which the pegs are inserted into the target holes. To increase the stringency of the NHPT, we modified and standardized the test, which we henceforth refer to as the Standardized Nine Hole Peg test (S-NHPT). The experimental setup with two pegboards was in analogy with that of a study exploring three different methods of completing the NHPT, focusing on comparisons to tests in a virtual setting [12]. However, we have standardized the experimental setup even further by stipulating the order in which the pegs should be transferred.

Kinematic assessments may detect changes in movement performance that are not captured by only considering the time taken to complete the NHPT [14], and provide objective measures that may be more sensitive and not vulnerable to ceiling effects [16]. Recent research calls for parameters indicating quality of movements in persons post-stroke by means of kinematic analysis in order to better understand motor recovery [14, 15, 17]. However, a test of fine upper limb fine dexterity like the NHPT has not been investigated. Our modifications and standardization enabled our first aim to kinematically characterize S-NHPT performance in a group of persons post-stroke and compare it to that of a non-disabled control group (discriminant validity). A second aim was to determine the convergent validity of the S-NHPT by comparing kinematics (movement time, peak speed, number of movement units, reach-grasp ratio, path ratio, acromion vertical displacement and trunk displacement) to the total movement time and to other clinical assessments (the Fugl-Meyer Assessment, the Stroke Impact Scale and grip strength). A third aim was to establish the within-session reliability of the S-NHPT, i.e., the consistency of the hand trajectories during the nine pick-up and transfer movements of the test.

## Methods

### Participants

Thirty persons post-stroke (69 ± 9 (SD) years, 19 men, 22 ± 17 months since onset) and 41 non-disabled controls (66 ± 12 years, 22 men) participated (Table 1). The stroke group was recruited from two clinics and met the following criteria: (a) adults aged 35–85 years old, (b) residual unilateral hemiparesis following an ischemic or hemorrhagic stroke at least 3 months previously, (c) discharged from the clinic and judged medically stable, (d) able to voluntarily lift both hands to nose, (e) able to understand verbal and written information, and (g) no impairments or diseases other than stroke that influenced the upper limb movements. The controls did not have musculoskeletal or neurological movement problems and were recruited among staff, acquaintances and through an organization for retired persons. Participants provided their written informed consent according to the Declaration of Helsinki and the study was approved by the Regional Ethical Review Board in Umeå, Sweden (dnr 2011–199-31 M).

**Table 1:** Participant Characteristics^a^

Characteristic	Stroke group (*n* = 30)	Control group (*n* = 41)
Sex (man/woman), n	19/11	22/19
Age, years	69 (9)	66 (12)
Body Mass Index, kg/m^2^	27.9 (2.9)	24.8 (2.2)*
Grip strength, kg (aff/non-dom)	26.3 (10.0)	35.2 (9.2)*
Grip strength, kg (non-aff/dom)	33.6 (9.5)	36.6 (9.6)
Handedness (right/left), n	29/1	39/2
Time since stroke, months	22 (17)	
Side of paresis (right/left), n	13/17	
Etiology (infarct/hemorrhage), n	26/4	
FMA UE (0–66)	54 (7)	
≥50 (mild)/≤49 (moderate), n	23/7	
Hypoesthesia/dysesthesia, yes/no	11/19	
Impaired thumb position, yes/no	2/28	
Spasticity in the affected arm, yes/no	9/21	
SIS (normalized score 0–100)		
Strength	66 (13)	
Hand function	76 (17)	
Mobility	88 (11)	
ADL and IADL	88 (13)	
Physical domain	79 (10)	

^a^Measurements are reported as mean (standard deviation) unless otherwise reported. *FMA UE* upper extremity part of the Fugl-Meyer Assessment (maximal score 66), *aff* affected arm (stroke), *non-dom* non-dominant arm (control), *SIS* Stroke Impact Scale (maximum score 100); IADL, Instrumental Activities of Daily Living. *Significant difference

### Clinical assessments

Motor impairment was evaluated with the *Fugl-Meyer Assessment for the upper extremity* (FMA-UE) [18]. The 33-item scale consists of three response categories (scores 0–2) for each item, with a maximum score of 66, indicating no impairment. The stroke group scored between 39 and 64 on the FMA-UE (Table 1), and were considered to have mild to moderate motor impairments [19]. In addition, sensory function was assessed with a subscale of FMA-UE, where a total score of 12 corresponds to no impairment. Sensation was assessed with eyes closed and in comparison to the unaffected side [18]. Light touch was tested for the arm and palmar surface of the hand (score 0 = anesthesia, 1 = hypoesthesia or dysesthesia, and 2 = normal. Maximum score = 4). Joint position sense was tested for the thumb (IP-joint), wrist, elbow and shoulder (score 0 = less than ¾ of the answers correct or absence), 1 = ¾ of the answers correct or considerable difference, 2 = correct 100%, little or no difference. Maximum score = 8). Two stroke participants had decreased proprioception of the thumb while eleven stroke participants had hypoesthesia/dysesthesia on the palmar surface of the hand.

The *Modified Ashworth Scale* (MAS) [20] was used to assess increased resistance to passive movement, in this paper for simplicity referred to as spasticity in analogy with what is commonly used in clinical settings. Muscle tone was tested for shoulder adductors, elbow flexors, wrist flexors, and finger flexors in a resting position on a 6-point ordinal scale ranging from 0 (no increase in muscle tone) to 4 (affected part rigid in flexion or extension) [20]. A MAS score of ≥1+ in one or more muscles indicated spasticity, which was found in nine persons post-stroke.

The stroke group completed the *Stroke Impact Scale* (SIS) [21] to assess their difficulties after their stroke. The SIS contains eight dimensions: strength, hand function, mobility, activities of daily living, emotion, memory, communication, and social participation. The first four dimensions create a physical domain. The SIS is an ordinal scale with five response categories (scores 1–5), and the scores produce a number between 0 and 100, with 100 indicating normal function [22]. For this study, only the hand function domain was analyzed (from now on referred to as SIS-Hand), which includes activities such as carrying heavy objects, turning a doorknob, opening a can or jar, tying shoelaces, and picking up a dime. SIS-Hand scores ranged between 30 and 100 with an average of 76, indicating a relatively large variation in perceived difficulties with hand function.

*Grip strength* was measured with a digital hand dynamometer (Jamar®, US), and taken as the mean of three trials. Grip strength was approximately 25% lower in the affected hand of participants post-stroke compared to the non-dominant hand of controls. The first author (GMJ) performed all the clinical assessments.

### Experimental procedure

**Fig. 1:**
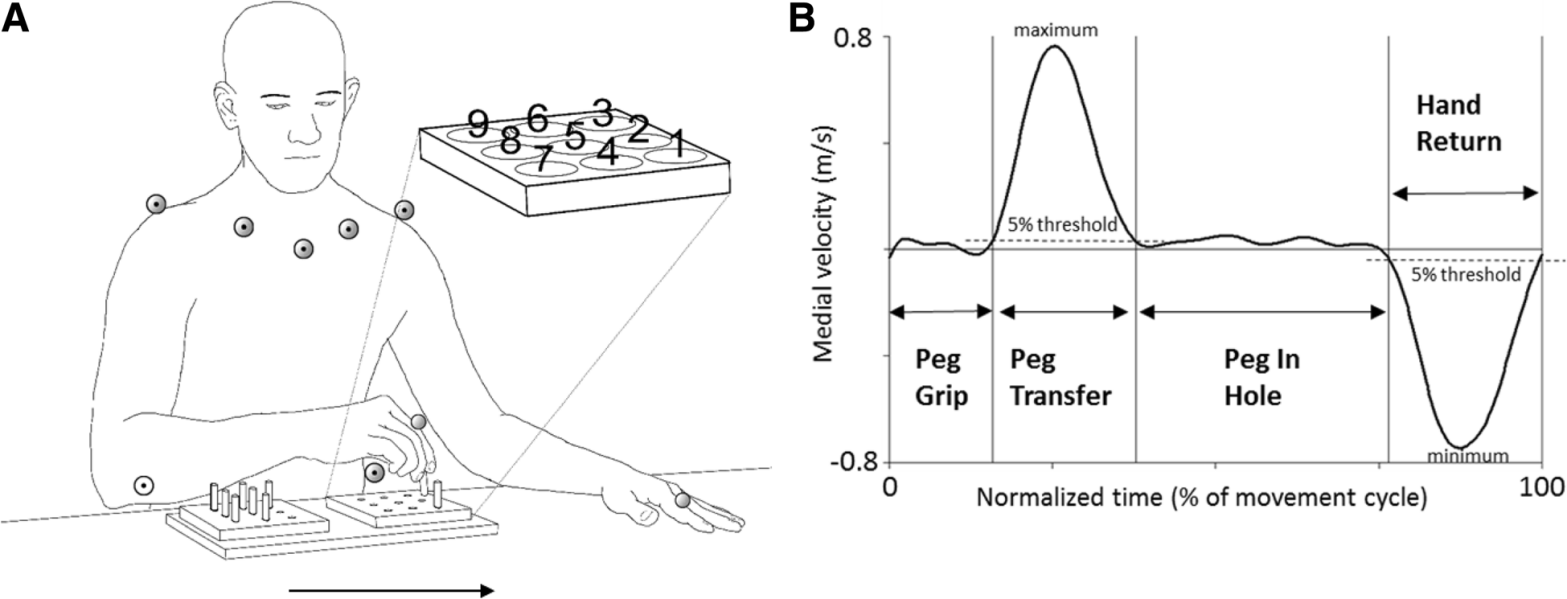
Experimental setup and movement phases. **a**) Marker positions used for the calculations of the kinematic variables. Markers displayed with a dot in the center of the marker were positioned on the trunk. The enlarged pegboard shows the standardized order of which peg to pick and which hole to fill, referred to as the “vertical row strategy”. The S-NHPT consists of 9 pegs (3.8 cm long, 0.64 cm wide) and two pegboards (12.7 cm × 12.7 cm) with 9 holes (0.70 cm wide) spaced 3.2 cm apart. The two pegboards were attached to a wooden panel with a distance of 18 cm between the center holes of the pegboards. The arrow indicates the direction of the movement. **b**) The velocity of the index finger marker in the medial direction displays the events defining the transfer phases Peg Transfer (positive curve) and Hand Return (negative curve). The manipulative phases Peg Grip and Peg In Hole are between those transfer movements (see Methods)

The participants were seated with their back supported but not restrained in a height-adjustable chair (Mercado Medic REAL® 9000 PLUS) that was attuned to each participant. Theirs arms rested on a table (height 74.5 cm) in front of them with elbows in 90° flexion and palms downward. The new combined double pegboard was positioned between their arms on the table with the empty medial pegboard placed at the participant’s midline and the peg-filled lateral pegboard oriented towards the hand being tested (Fig. 1). Pegs from the lateral pegboard were picked up unimanually one at a time, transported and inserted into the holes of the medial pegboard and then returned likewise one by one to the lateral pegboard. The participants were instructed to perform the test as quickly and as accurately as possible according to the specific order of the “vertical row strategy” described in Fig. 1. No more attempts were allowed if they did not follow the specific order. If a peg was dropped on the table or knee, the participant was allowed to pick it up. If the peg was dropped on the floor, the participant continued with the next peg. The test performance was verified by video recording. After a familiarization trial, the S-NHPT was repeated twice for both hands. The number of trials was limited to the 1 + 2 protocol due to time restrictions and to avoid fatigue in the stroke group because additional movement tests were also performed in the same test session as part of a larger data collection. The stroke group performed the test 1 + 2 with the non-affected arm and then 1 + 2 with the affected arm, while the control group started with their dominant arm.

Upper limb and trunk motions during the S-NHPT test were recorded using an 8-camera motion capture system (240 Hz, Oqus®, Qualisys AB Gothenburg, Sweden). Nine passive retroreflective markers were affixed on specific anatomical landmarks; index finger, acromion, clavicula, sternum and crista iliaca at the most superior and lateral point of the crest (for full marker set see Fig. 1). Two digital video cameras (Canon Legria HV40), integrated with the motion analysis system, recorded movements in the sagittal and frontal planes. Data were collected in Qualisys Track Manager (QTM, version 2.6; Qualisys, Gothenburg, Sweden), and exported to Visual 3D (version 5, C-motion Inc., Germantown, MD, USA). All data were filtered with a 6 Hz Butterworth filter prior to calculations.

### Data analysis

The S-NHPT, like the original NHPT, consists of two sequential tasks in opposite movement directions: A) *Medial Peg Transfer* (transfer of all the pegs from the lateral to the medial pegboard into the holes) and B) *Lateral Peg Return* (return of all the pegs from the medial to the lateral pegboard into the holes). In the present study, only the best trial of the first task Medial Peg Transfer was analyzed. The best trial was defined as the one in which less pegs were dropped, or in a few cases determined by obscured or lost markers. The rationale for analyzing only one trial was based on the fact that we wanted to have as good performance as possible for a stable kinematic evaluation, and also avoid averaging across trials that would mask potential interrelated movement aspects. This trial was divided into four consecutive movement phases: *Peg Grip*, *Peg Transfer*, *Peg In Hole* and *Hand Return* for each peg transfer (Fig. 1). The beginning/end of the Peg Transfer were defined as the time point at which the velocity of the index finger marker in medial direction exceeded/fell below a threshold set to 5% of the *maximal* value, and remained above/below this value for at least 40 ms. The beginning/end of the Hand Return were defined as the time point when the velocity of the index finger marker in the medial direction exceeded/fell below 5% of the *minimal* value, and remained above/below it for at least 40 ms. The *Peg Grip* and *Peg In Hole* were manipulative phases between those transfer movements (Fig. 1).

The markers on each index finger were used to analyze hand motions, and acromion markers were used to calculate vertical acromion displacement. The markers on the upper body (including acromion markers) were used to calculate the segment angle of the trunk based on the Visual3D six degrees of freedom model. The local frames of the trunk were defined so that +X corresponded to flexion, +Y to adduction and + Z to internal rotation, and the angles were calculated using Cardan angles (X-Y-Z sequence), following the Joint Coordinate System by Cole et al. [23], which also follows ISB recommendations [24].

We calculated the time of the first task (A) of the S-NHPT, from now on referred to as *Total Movement Time* (TMT), and the time for each of the four sub-phases for each peg transfer. *Grasp-Reach ratio* was computed as the ratio of the time spent in manipulative phases (Peg Grip and Peg In Hole) compared to transfer phases (Peg Transfer and Hand Return) of the task. *Peak speed* (mm/s) was defined as the maximum tangential velocity that the index finger obtained during the Peg Transfer and Hand Return, respectively, while the *Time to Peak speed* (TPS) indicated the time from movement onset until maximum detected speed from the acceleration curves and was expressed in seconds. Movement smoothness was quantified by computing the *Number of Movement Units* (NMU) of the index finger marker by calculating the number of local maxima in the tangential velocity curve [25, 26]. A cut-off value corresponding to > 10% of the peak velocity was used to accept an event in order to avoid erroneously detecting movement units when no movements occurred. According to this definition, a smooth movement would have only one movement unit. Movement efficiency, or straightness, was estimated by the *Path ratio*, which is the ratio of the distance of the actual movement path and the path distance of an ideal straight line. The path ratio of the index finger was calculated for the transfer phases Peg Transfer and Hand Return. To identify possible compensatory movements in adjacent body segments, the *Trunk displacement* and *Acromion displacement* were calculated. The Trunk displacement was defined as the difference between the highest and lowest range of motion value for thoracic rotation during Peg Transfer and Hand Return, respectively. The Acromion displacement was computed as the displacement of the acromion marker in the vertical direction during the entire test from initial position (when the hand was placed on the table) until end position (pegs returned to the lateral peg board and hand returned back on the table). It represents a global measure of frontal plane excursion that involves scapular movements relative to the trunk as well as trunk movements. Positive values indicated displacement in the cranial direction while negative values denoted displacement in the caudal direction.

Comparable movement trajectories were achieved for all participants except for two persons post-stroke who were not able to perform the test with the requested peg order in either of the two attempts. We compared the kinematics between the non-dominant arm of the control group and the affected arm of the stroke group, since movement kinematics of the non-dominant arm of non-disabled controls might be more evenly matched with those of the affected arm. To test the possibility that any differences in movement parameters within the stroke group might have been due to hand dominance before stroke onset, we compared persons who were affected in their dominant arm (*n* = 14) and persons who were affected in their non-dominant arm (n = 14). Further, to evaluate the effects of sensory impairments within the stroke group, we compared persons with impaired light touch (*n* = 11) to persons with no detectable sensory impairments (*n* = 17). Finally, to evaluate possible effects of spasticity within the stroke group, we compared persons with spasticity (*n* = 8) to persons with no spasticity (*n* = 20).

### Statistical analysis

The statistical analysis was performed with IBM SPSS (Statistical Packages for Social Sciences, 21.0) with a chosen significance level of 0.05. Comparisons between groups and between subgroups were made with independent *t* tests or the Mann-Whitney U test, depending on the data distribution. The *z* values of the nonparametric test was used to calculate effect sizes where *r* was interpreted as ≥0.5 = large, 0.3 to 0.5 = medium, and ≤ 0.1 = small effect sizes [27]. The relationships between kinematic variables and clinical assessments were estimated with Spearman’s correlation coefficient. The strength of correlation was interpreted according to Munro: 0.00–0.25 = little if any, 0.26–0.49 = low, 0.50–0.69 = moderate, 0.70–0.89 = high and ≥ 0.90 = very high correlation [28]. For the within-session reliability, nine movement trajectories of the best trial were analyzed. The reliability was evaluated with the Intraclass correlation coefficient (ICC_3,k_) and the standard error of measurement (SEM = SD_TEST_ x (1-ICC)) with SD_TEST_ calculated from all the completed trials [29].

## Results

### Discriminant validity of the S-NHPT

**Fig. 2:**
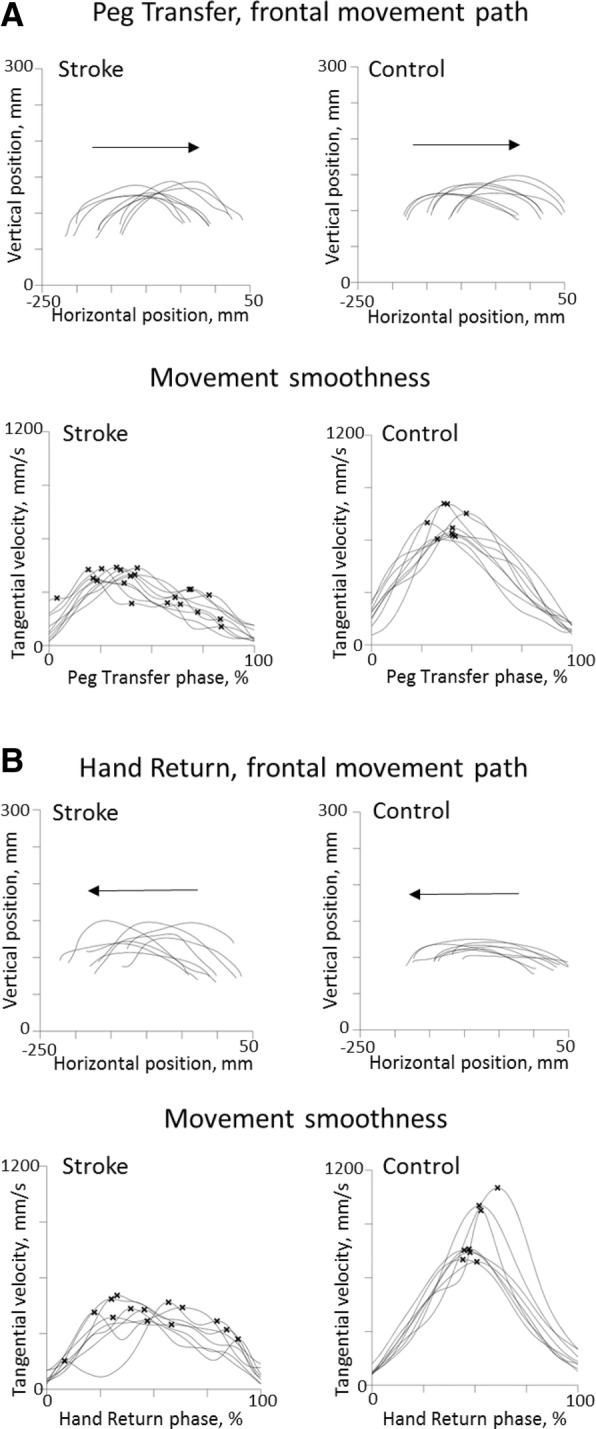
Movement paths from the stroke group and the control group. Examples of movement paths of the markers of the index finger in the frontal plane, and velocity profiles with marked number of movement units of one person post-stroke (left panel) and one control person (right panel) for the Peg Transfer Phase (**a**) and Hand Return Phase (**b**), respectively. The arrows indicate the direction of the movements

Twenty-eight persons with stroke and 41 controls were able to perform the test in the requested order. The stroke group performed the S-NHPT significantly slower than controls (TMT mean difference = 9.8 s, SE mean 1.4), as shown in Table 2. The mean difference between groups for Peg Grip time was 0.37 s, Peg Transfer time 0.17 s, Peg In Hole time 0.44 s, and for Hand Return time it was 0.15 s. The Peak speed for both Peg Transfer and Hand Return were slower for the stroke group (mean difference 17.2 s and 15.7 s, respectively). Likewise, the stroke group needed a longer time to peak speed for both Peg Transfer and Hand Return (mean difference TPS 6.6 s and 5.6 s, respectively). The Grasp-Reach ratio was 1.18 for the stroke group and 0.73 for the control group, indicating that the stroke group spent a much longer relative time in the manipulative phases than in the transfer phases. The stroke group also had less smooth movements (mean difference NMU Peg Transfer = 0.40 and Hand Return = 0.40) compared to controls. For the spatial variables; the stroke group demonstrated slightly increased Trunk displacement (Peg Transfer mean difference = 1.4°, and Hand Return mean difference = 1.6°) and increased Acromion vertical displacement during the entire test (mean difference = 15.7 mm) compared to the control group. The stroke group also had an increased Path ratio during Hand Return (mean difference 0.03). Figure 2 shows examples of movement paths with increased variability and increased NMU for a typical post-stroke individual in the subgroup moderate stroke compared to a control person selected based on average measures. The stroke group also had lower hand strength in the affected arm in comparison to the non-dominant arm of the control group (*p* < 0.01) as shown in Table 1.

**Table 2:** Kinematic outcomes of the Standardized Nine Hole Peg test in all participants^a^

Variables	Stroke group (*n* = 28) affected arm	Control group (n = 41) non-dominant arm	*p*-value	Effect size
Total movement time, s	22.42 (8.57)	12.66 (1.97)	< 0.001	0.72
Time for Peg Grip, s	0.59 (0.33)	0.22 (0.09)	< 0.001	0.72
Time for Peg Transfer, s	0.61 (0.15)	0.44 (0.07)	< 0.001	0.65
Time for Peg In Hole, s	0.85 (0.55)	0.40 0.12)	< 0.001	0.61
Time for Hand Return, s	0.57 (0.1)	0.42 (0.05)	< 0.001	0.58
Grasp-Reach ratio	1.18 (0.57)	0.73 (0.21)	< 0.001	0.57
Peak speed Peg Transfer, s	0.59 (0.13)	0.76 (0.10)	< 0.001	0.61
Peak speed Hand Return, s	0.71 (0.17)	0.87 (0.09)	< 0.001	0.48
Time to peak speed Peg Transfer, s	0.23 (0.05)	0.17 (0.02)	< 0.001	0.68
Time to peak speed Hand Return, s	0.29 (0.08)	0.23 (0.03)	< 0.001	0.42
NMU for Peg Transfer, n	1.6 (0.5)	1.1 (0.2)	< 0.001	0.57
NMU for Hand Return, n	1.5 (0.4)	1.1 (0.1)	< 0.001	0.44
Path ratio Peg Transfer	1.24 (0.08)	1.21 (0.04)	NS	
Path ratio Hand Return	1.17 (0.09)	1.12 (0.04)	< 0.001	0.32
Trunk displacement for Peg Transfer, °	3.5 (2.0)	2.1 (0.5)	< 0.001	0.50
Trunk displacement for Hand Return, °	3.8 (2.5)	2.3 (0.5)	< 0.001	0.43
Acromion displacement, mm	37.9 (19.3)	22.2 (9.3)	< 0.001	0.42

^a^Results are reported as mean (standard deviation). *NMU* number of movement units

### Subgroup analysis

The analysis of right and left handed persons post-stroke did not reveal any significant relationship between kinematic outcomes and hand dominance before stroke. The subgroup with sensory impairment in the affected hand had increased Acromion displacement compared to the subgroup without sensory impairments (*p* < 0.05 and effect size = 0.39). Persons post-stroke who had spasticity performed the transfer phases (Peg Transfer and Hand Return) slower and with increased Trunk displacement compared to the persons post-stroke without spasticity (*p* < 0.01 and effect sizes = 0.51–0.55).

### Convergent validity of the S-NHPT

For the stroke group (Table 3), NMU correlated moderately or highly with TMT (Peg Transfer, *r*_*s*_ = 0.58 and Hand Return *r*_*s*_ = 0.77) and FMA-UE (*r*_*s*_ = 0.60). Spatial variables were weakly correlated with TMT (Trunk displacement for Peg Transfer, *r*_*s*_ = 0.39 and Hand Return, *r*_*s*_ = 0.38, Acromion displacement, *r*_*s*_ = 0.42, Path ratio, *r* = 0.32), as shown in Table 3. Acromion displacement was likewise weakly correlated to clinical outcome measures (FMA-UE, *r*_*s*_ = 0.45 and SIS-hand, *r*_*s*_ = 0.38). The FMA-UE was the only clinical assessment whose outcome correlated significantly with more than one kinematic variable of the S-NHPT.

**Table 3:** Correlations between kinematic variables of the Standardized Nine Hole Peg test and clinical tests

	TMT	NMU PT	NMU HR	TD PT	TD HR	AD	FMA-E	Grip strength
Total movement time								
NMU for PT	0.583**							
NMU for HR	0.767**	0.563**						
Trunk displacement for PT	0.394*	0.412*	0.355					
Trunk displacement for HR	0.376*	0.383*	0.332	0.934**				
Acromion displacement	0.417*	0.380*	0.621**	0.572**	0.618**			
FMA-UE	−0.595**	−0.368	−0.412*	−0.423*	−0.442*	−0.445*		
Grip strength	−0.360	−0.131	−0.223	−0.157	−0.113	−0.226	0.283	
SIS-hand	− 0.117	− 0.326	− 0.233	−0.287	− 0.350	−0.381	0.179	−0.032

*TMT* Total Movement Time, *NMU* Number of movement units, *PT* Peg Transfer, *HR* Hand Return, *TD* Trunk displacement, *AD* Acromion displacement, *FMA-UE* Fugl-Meyer Assessment for upper extremity, *SIS-hand* Stroke Impact Scale hand domain. *p < 0.05, **p < 0.01

### Within-session reliability of the S-NHPT

For both groups, the within-session reliability of the S-NHPT was generally high to very high (ICC 0.71–0.97, Table 4). For the stroke group, the Time for Peg in Hole had the lowest within-session reliability (ICC = 0.43 and SEM = 2.37). For the control group, the NMU for Hand Return showed the lowest within-session reliability (ICC = 0.37 and SEM = 0.66).

**Table 4:** Within-test reliability of the Standardized Nine Hole Peg Test

Variables	Stroke group (n = 28) affected arm		Control group (n = 41) non-dominant arm	
	ICC (95% CI)	SEM	ICC (95 CI)	SEM
Time for Peg Grip, s	0.80 (0.66–0.89)	0.51	0.73 (0.59–0.84)	0.18
Time for Peg Transfer, s	0.92 (0.87–0.96)	0.10	0.90 (0.84–0.94)	0.06
Time for Peg In Hole, s	0.43 (0.05–0.70)	2.37	0.71 (0.55–0.83)	0.29
Time for Hand Return, s	0.97 (0.94–0.98)	0.04	0.91 (0.86–0.95)	0.04
Peak speed Peg Transfer, s	0.96 (0.93–0.98)	0.05	0.94 (0.90–0.96)	0.05
Peak speed Hand Return, s	0.97 (0.94–0.98)	0.06	0.91 (0.86–0.94)	0.11
Time to peak speed Peg Transfer, s	0.44 (0.07–0.71)	0.26	0.65 (0.46–0.79)	0.06
Time to peak speed Hand Return, s	0.89 (0.81–0.94)	0.07	0.85 (0.76–0.91)	0.04
NMU for Peg Transfer, n	0.78 (0.62–0.88)	0.93	0.61 (0.40–0.77)	0.60
NMU for Hand Return, n	0.81 (0.69–0.90)	0.66	0.37 (0.03–0.63)	0.66
Path ratio Peg Transfer	0.44 (0.07–0.70)	0.51	0.82 (0.72–0.89)	0.07
Path ratio Hand Return	0.85 (0.75–0.92)	0.10	0.91 (0.87–0.95)	0.03

*NMU* Number of movement units, *ICC* Intraclass correlation coefficients, *SEM* standard error of measurement

## Discussion

Our innovative modification and standardization of the NHPT made it possible to kinematically quantify motor performance during four consecutive movement phases of the test by calculating temporal and spatial movement parameters.

### Discriminant validity of the S-NHPT

As expected, the time to perform the S-NHPT was prolonged for the stroke group compared to controls, which is also consistent with a recent study for performance of the NHPT [30]. Further, the kinematic measures based on phase analysis showed that our stroke group spent a significantly longer time grasping and releasing pegs in the manipulative phases relative to the transfer phases of the task compared to controls. This emphasized the decreased dexterity among persons post-stroke despite moderately or even mildly affected hand dysfunction. Our controls took on average 1.5 s less to complete the S-NHPT than another healthy group took to complete a modified NHPT in a study by Bowler et al. [12]. Although both protocols required inserting nine pegs, the different movement due to pegboard placements, or potential differences in distance between the pegboards (information not provided in [12]), or task instructions likely explain the observed time difference across studies.

In agreement with previous studies of other upper limb tasks such as reach-to-grasp a ball, drink from a glass, and point without vision to a remembered target [16, 26, 31], we found that movement smoothness, quantified as NMU, clearly discriminated persons post-stroke from non-disabled controls. Previous studies have reported compensatory upper body movements post-stroke such as altered scapulohumeral rhythm during tasks involving shoulder flexion [32, 33], and increased anterior trunk movements during forward reaching [19, 26, 34]. Indeed, our stroke group also demonstrated compensatory scapular/trunk movements during the S-NHPT which would obviously not be captured by the original NHPT. This compensation may be due to the fact that persons post-stroke tend to produce concurrent flexion motions in the shoulder and elbow joints when attempting any movement in the upper limb [35], resulting in increased motion in the scapular/trunk segments. An increased notion of and possibility to quantify compensatory movement patterns in the clinics would most likely be beneficial for clinical analysis, treatment and evaluation.

### Subgroup analysis

Handedness has been suggested to have little functional impact on time to perform the NHPT in non-disabled persons [3]. This was supported by Bowler et al. [12], who found that hand dominance had no impact on healthy persons’ time to perform three different ways of completing NHPT. However, it was recently shown that persons in the subacute phase post-stroke performed the NHPT with a lower velocity (pegs/s) if the affected hand was the non-dominant hand before stroke [30]. In our study however, hand dominance before stroke showed no influence on S-NHPT performance. This might imply that S-NHPT is not as sensitive to hand dominance, perhaps because it requires less fine manipulative skills (to a larger degree involving most of the fingers) than the NHPT. However, the impact of hand dominance on Virtual Peg Insertion Test performance was not addressed in persons post-stroke [13] nor persons with multiple sclerosis [36]. In any case, it would have been difficult to make a comparison with the S-NHPT performance since the VPIT requires other components of upper limb motor performance than the NHPT [13]. Sensory impairments did not influence temporal aspects of the S-NHPT either, at least not in our somewhat limited study population with sensory reductions. Furthermore, the persons with sensory impairments and persons with spasticity used excessive scapular/trunk movements as indicated by increased acromion displacement and increased trunk displacement, respectively. This is in line with previous studies which have shown that sensory deficits correlate with abnormal reaching performance [35] and that spasticity complicates NHPT performance [8].

### Convergent validity of the S-NHPT

To investigate the relationship between kinematics (e.g. movement time, smoothness and compensation) and clinical assessments, the TMT was chosen as a representative temporal variable since all variables based on time or speed were correlated to each other. The correlation between the TMT and NMU was moderate to high for the transfer phases (Peg Transfer *r*_*s*_ = 0.58 and Hand return 0.77). An even higher correlation between TMT and NMU (*r*_*s*_ = 0.86) has been reported for a drinking task, where the authors suggested that TMT may be used as an indirect measure of movement smoothness [37]. However, movement speed was not controlled for. The effect of speed on NMU was investigated in another study, where reduced movement speed did not fully explain the increased temporal segmentation in persons post-stroke [31]. Within our stroke group, the TMT also correlated, albeit modestly, with the FMA-UE (*r*_*s*_ = 0.60). A low-to-fair correlation between the total time of the NHPT and FMA-UE (*r*_*s*_ = 0.27) has been reported by Lin and co-workers [7], where the TMT of the NHPT was highly variable (137 ± 99 s) for the stroke population who had mild-to-moderate impairments (FMA 51 ± 7 scores). However, this result may not be directly comparable to our study since we calculated the TMT of the S-NHPT based on the first sequential task (Medial Peg Transfer), while TMT of the NHPT traditionally include the return phase (Lateral Peg Return). In the study by Lin et al., the high variation of TMT was nevertheless probably due to the none-standardized procedure of the NHPT, which resulted in a lower correlation between the TMT and the FMA-UE compared to our study where we used the novel S-NHPT. The Acromion displacement was moderately correlated with the Trunk displacement, indicating that these movements usually increase simultaneously during the NHPT in persons post-stroke. However, as the trunk motion ranges were only a few degrees, thoracic rotation may not be crucial in this test. Neither grip strength, nor SIS-hand correlated with the S-NHPT, which is contrary to other studies using the NHPT [1, 7, 10, 38, 39]. This might be because the persons post-stroke in those studies had a wider range of upper limb impairments than those in the present study.

### Within-session reliability of the S-NHPT

The within-session reliability of most kinematic variables was moderate to very high for both groups. For both groups, the time of the manipulative phases were less reliable (lower ICCs and higher SEMs) than the transfer phases. Particularly the stroke group failed to repeat consistent movements during the Peg In Hole phase. The latter result is not surprising since the peg-in-hole insertion implies a more challenging dexterous task than the other phases. In a study that investigated the test-retest reliability of the computerized VPIT in persons post-stroke [13], the execution time and the NMU showed better reliability (ICC 0.83–0.94) than the path ratio (ICC 0.67–0.70). Although these results are not directly comparable to ours, some of the reliability estimates were similar for movement time, NMU and path ratio. No other study prior to ours, however, seems to have investigated within-session reliability of any version of NHPT post-stroke. We chose to analyze the best trial, and there seemed not to be any obvious learning effects across the two trials, as the first trial was considered as the best in the majority of cases (for 16 patients and 25 controls).

### Clinical implications

The protocol implemented in the present paper, with a motion capture system and full upper-body marker set, requires more time and resources than what is realistic for most clinical settings. Our intention with this work was not to suggest that complex setups with 3D motion capture should always be used in the clinics but rather to pave the way for development of future more hands-on tests in which simplified marker sets or marker-free techniques are used in combination with, for instance, video-based motion capture tools/apps. However, this can only be done once more thorough approaches like our own have identified which crucial movement aspects to focus on and revealed the relationships between these, as well as clarified what various movement parameters represent. The S-NHPT per se is not more complicated than the original test and may be used as a timed peg test that provides comparable measures by combining two pegboards from two original tests (see Fig. 1). It is possible to implement with or without motion analysis tools. In contrast to the original NHPT, the proposed S-NHPT has the benefit of enabling direct comparison, i.e. also curve overlays of kinematic movement trajectories provided also by video in combination with less complex motion analysis software tools when sophisticated 3D systems are not available. The specific order of which peg to pick and which hole to fill in the S-NHPT may however impose greater cognitive demands on memory and attention than during the self-guided performance in the original test. Indeed, two persons in our stroke group were excluded as they could not manage the specific order of the S-NHPT. As most daily activities involve elements of cognition during movements, it has been suggested that these movement and cognitive aspects should not be assessed or treated separately in persons with brain injuries [40]. Therefore, retaining the specific order of pegs in the S-NHPT is justified as it may detect interference between cognition and dexterity that is otherwise not possible in the NHPT.

### Limitations

The generalizability of this study is limited to persons post-stroke with mainly mild impairments. The sample size was considered sufficient for group comparisons according to our power analysis. For the subgroup analysis, however, it would have been preferable to have had larger samples. Another limitation was that cognition was not assessed, so it could not be analyzed in relation to movement performance, which remains a scope for future studies. Further, we did not control for speed regarding the differences in kinematic variables between the stroke group and the control group. However, we do not believe that the differences in excessive scapular/trunk movements were only explained by the fact that the stroke group performed the S-NHPT slower than the control group. In fact, in a parallel study of the timed Finger-to-Nose Test in the same study population and based on data collection at the same test occasion, we found consistent differences in scapular/trunk movements in spite of equivalent speeds [41]. Another consideration is that the S-NHPT involves initially picking up the pegs from a pegboard rather than a container as in the NHPT, and thus requires a pinch grip that requires less fine manipulative control than from a pile of intermingled pegs. This modification of the test implies more focus on a precision grip rather than in-hand manipulation. However, this likely results in enabling more persons, even with moderate impairments, to perform the S-NHPT than the original test. It would in this context have been optimal to directly compare the standardized test to the original NHPT. However, this was not possible in the present study since it is part of a larger project comprising 3D data collection to evaluate several upper limb tasks and also gait performance in our study population. Due to time limitations for the test session and to not further increase fatigue in persons post-stroke, the number of tests and repetitions of each test were limited.

## Conclusions

The modification and standardization of the NHPT shows adequate discriminant validity, convergent validity and within-session reliability, and also enables kinematic analysis of movement performance. Future research should ascertain further psychometric properties, e.g. sensitivity, of the S-NHPT. Kinematic evaluation offers added value to current time-based measures through a more comprehensive assessment of dexterity post-stroke that identifies differences in movement performance which are not captured in the NHPT.

## Abbreviations

FMA-UE: Fugl-Meyer Assessment scale for the Upper Extremity
ICC: Intraclass Correlation Coefficients
MAS: Modified Ashworth Scale
NHPT: Nine Hole Peg test
NMU: Number of Movement Units
SEM: Standard Error of Measurement
SIS: Stroke Impact Scale
S-NHPT: Standardized Nine Hole Peg test
TMT: Total Movement Time
TPS: Time to Peak Speed
